# Bicomponent Electrospinning of PVDF-Based Nanofiber Membranes for Air Filtration and Oil–Water Separation

**DOI:** 10.3390/polym17050703

**Published:** 2025-03-06

**Authors:** Tianxue Feng, Lin Fu, Zhimei Mu, Wenhui Wei, Wenwen Li, Xiu Liang, Liang Ma, Yitian Wu, Xiaoyu Wang, Tao Wu, Meng Gao, Guanchen Xu, Xingshuang Zhang

**Affiliations:** 1 Advanced Materials Institute, Qilu University of Technology (Shandong Academy of Sciences), Jinan 250014, China; 2 Sino Science and Technology Co., Ltd., Dongying 257000, China; 3 Guochen Industrial Group Co., Ltd., Jinan 250300, China

**Keywords:** electrospinning, PVDF, nanofiber membrane, air filtration, oil–water separation

## Abstract

Particulate matter (PM) and water pollution have posed serious hazards to human health. Nanofiber membranes (NFMs) have emerged as promising candidates for the elimination of PMs and the separation of oil–water mixtures. In this study, a polyvinylidene difluoride (PVDF)-based nanofiber membrane with an average diameter of approximately 150 nm was prepared via a double-nozzle electrospinning technology, demonstrating high-efficiency PM filtration and oil–water separation. The finer fiber diameter not only enhances PM filtration efficiency but also reduces air resistance. The high-voltage electric field and mechanical stretching during electrospinning promote high crystallization of β-phase PVDF. Additionally, the electrostatic charges generated on the surface of β-phase PVDF facilitate the adsorption of PM from the atmosphere. The introduction of polydopamine (PDA) in PVDF produces abundant adsorption sites, enabling outstanding filtration performance. PVDF-PVDF/PDA NFMs can achieve remarkable PM_0.3_ filtration efficiency (99.967%) while maintaining a low pressure drop (144 Pa). PVDF-PVDF/PDA NFMs are hydrophobic, and its water contact angle (WCA) is 125.9°. It also shows excellent resistance to both acidic and alkaline environments, along with notable flame retardancy, as it can self-extinguish within 3 s. This nanofiber membrane holds significant promise for applications in personal protection, indoor air filtration, oily wastewater treatment, and environmental protection.

## 1. Introduction

With the rapid development of the economy and urbanization, issues related to urban pollution and protection are increasingly receiving attention. On the one hand, the main culprit for the serious impact of air pollutants on the brain is PMs with a diameter of less than 2.5 μm [1]. Especially, PM_0.3_ has a greater impact on human health due to its small particle size, attachment of large amounts of harmful and toxic substances, wide dispersion range, and long residence time in the atmosphere [2]. On the other hand, water pollution can contaminate drinking water sources, causing a wide array of health issues from gastrointestinal disorders to long-term exposure-related cancers. As a result, the development of highly effective filtration technologies has become an urgent and crucial task in the field of environmental and health sciences [3]. In the existing filtration, membrane filtration technology has the advantages of low energy consumption and simple operation [4], such as hot pressing membrane forming technology, gel technology, wet technology, etc. Micropores formed during the hot pressing membrane process permeate each other, and their affinity is insufficient. There are many problems in the gel membrane process, such as large loss and difficulty in preparing nanomembranes. The wet process can produce microporous membrane materials with high micropore curvature, poor environmental protection, and poor thermal stability. Electrospun nanofibers have characteristics of continuity, large aspect ratios, large specific surface area, high flexibility, mechanical properties, and adjustable fiber diameters [5]. Researchers have developed a variety of methods for fabricating structured nanofiber membranes, with the aim of improving the filtration and separation performance of fiber membranes [6]. For example, increasing the thickness of a fiber membrane [7], changing the network structure of fiber membranes [8], and adding a certain charge to a fiber membrane can make it. However, improvement in filtration efficiency often brings about drawback of increased pressure. Thus, the quest to obtain membranes featuring high filtration efficiency and low air resistance remains a significant challenge.

Fiber air filtration mechanisms include gravity effects, sieving, interception, inertial collision, Brownian motion, and electrostatic effects [9]. When PMs in the air pass through filters, electrostatic forces not only effectively attract charged particles but also capture PMs through electrostatic induction effects [10]. As a kind of self-polarizing electret material, PVDF is a highly nonlinear and structural organic polymer with certain piezoelectric properties. PVDF undergoes various mechanical, thermal, and electrical treatments, resulting in the formation of five polycrystalline structures: α, β, δ, ε, and γ, which can interconvert [11]. The β phase overall trans of phase molecular chain is combined with specific polarization in the crystal to produce the highest piezoelectric effect, accompanied by additional piezoelectricity [12]. More importantly, β-PVDF is formed under the stretching of the high-voltage electric field in the process of electrospinning. Polydopamine (PDA) contains a substantial number of catechol structures, endowing it with remarkable adhesion properties. Catechol structures can impart charges to nanoparticles. Owing to strong electrostatic repulsion force, nanoparticles encapsulated by PDA display extremely high stability. The strong adhesion enables PDA to adhere to the surface of inorganic and organic materials [13]. For example, Ma et al. [14,15] electrospun PVDF/PDA fiber membranes, presenting significant adsorption capacity for MB and Cu^2+^. Tian et al. [15] designed a two-stage electrostatically assisted air filtration device by a thin coating of PDA on the polyethylene terephthalate coarse filter. This filter achieved a high filtration efficiency of 99.48% for PM_0.3_. Therefore, PDA could be used to improve the adsorption capacity of membranes.

**Scheme 1:**
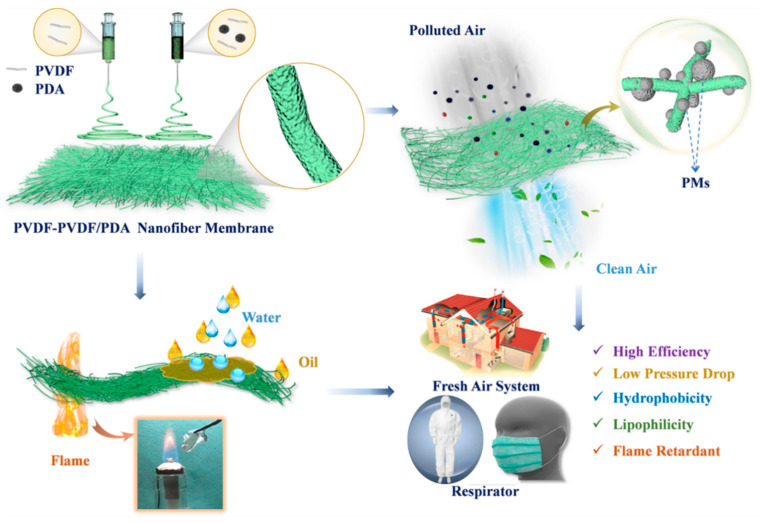
Illustration of the fabrication and properties of PVDF-PVDF/PDA NFMs.

Herein, we prepared PVDF-based nanofiber membranes featuring high PM filtration performance and efficient oil–water separation capabilities using a simple one-step double-nozzle electrospinning strategy (Scheme 1). The high-voltage electric field in electrospinning polarizes and stretches the PVDF jet, promoting β-PVDF high crystallization. The rich surface groups of PDA further enhance the filtration and separation performance of PVDF-PVDF/PDA nanofiber membranes (NFMs). Notably, the higher electronegativity of fluorine (F) atoms in PVDF compared to nitrogen (N) atoms in PDA induces electrostatic attraction, boosting the air filtration performance. At a basis weight of 8.82 g/m^2^, the PVDF-PVDF/PDA-3 NFMs achieved a PM_0.3_ filtration efficiency of 99.967% with a pressure of 144 Pa. Additionally, the hydrophobicity of PVDF-PVDF/PDA NFMs endows them with outstanding oil–water separation capabilities. Modified hydrophobic PVDF-PVDF/PDA NFMs also demonstrate remarkable acid/alkali resistance and flame-retardant properties. These characteristics make the membrane an ideal option for applications in personal protection during fire and other emergency situations, as well as in household air purification and filtration systems.

## 2. Material and Methods

### 2.1. Materials

*N*, *N′*-dimethylformamide (DMF, 99%, biotech grade), polyvinylidene difluoride (PVDF, M_w_: 400,000 g/mol, powder), dopamine hydrochloride, potassium hydroxide (KOH, 85%), ethanol (99.7%), ammonia (25~28%), NaCl, NaOH, dichloromethane (AR), and HCl were purchased from Shanghai Macklin Biochemical Co., Ltd., Shanghai, China. Ultrapure water was made in the laboratory.

### 2.2. Fabrication of Electrospinning PVDF/PDA and PVDF-PVDF/PDA NFMs

Preparation of PVDF NFMs: A total of 1.0 g of PVDF was dissolved in DMF and stirred for 4 h at 25 °C, forming a 15 wt% PVDF spinning solution. A 20 wt% PVDF spinning solution was prepared in the same way. PVDF spinning solutions were electrospun to form PVDF NFMs. The spinneret was 18 G, and the spinning voltage was 20 kV. The ambient temperature and humidity were 25 °C and 80 ± 5 °C. Finally, the obtained PVDF NFMs were dried at 60 °C for 24 h to remove residual solvent.

Preparation of PVDF-PVDF/PDA NFMs: A total of 0.1 g of PDA was dispersed and sonicated for 2 h in DMF. PVDF was added to the above solution, obtaining a PVDF/PDA-1 spinning solution. The 20 wt% PVDF and PVDF/PDA-1 spinning solutions were transferred into two 5 mL syringes, respectively. The two syringes were placed in the main and secondary pumps. Spinning parameters were the same as those described above. After spinning, nanofiber membranes were dried at 60 °C for 24 h, obtaining PVDF-PVDF/PDA-1 (labeled S-1) NFMs, as shown in Scheme 1. In the same method, 0.2 g, 0.3 g, and 0.5 g of PDA were added to prepare the spinning solution, obtaining PVDF-PVDF/PDA-x (x = 2, 3, 5), labeled as S-2, S-3, and S-5, respectively. To better illustrate the filtration properties of PVDF-PVDF/PDA-x NFMs, single-nozzle electrospun PVDF/PDA-x NFMs were prepared as a control group. For double-nozzle electrospinning, one nozzle contained PVDF while the other contained PVDF/PDA. Then, contrast experiments were performed by electrospinning using a single-nozzle PVDF/PDA.

### 2.3. Measurement and Characterization

The characterization methods for the physical and chemical properties of materials were presented in the Supporting Information (SI). The air filtration performance of PVDF NFMs was evaluated by filtration efficiency (*η*), pressure drop (*ΔP*), and quality factor (*QF*). Filtration efficiency and pressure drop of nanofiber membranes were measured using an automatic filtration material tester. Specifically, sodium chloride particle matters from 0.3 to 10 μm were selected as particle models for PMs. The ambient temperature of the tested nanofiber membrane was 25 ± 5 °C. The airflow velocity was controlled between 10 and 85 L/min. All tests were conducted three times. *QF* was calculated to describe the comprehensive air filtration performance of the air filter using the following equation [16]:

(1) $$QF=\frac{-ln(1-\eta )}{\Delta P}$$

where *η* is the filtration efficiency and Δ*P* is the pressure drop.

## 3. Results and Discussion

### 3.1. Morphology, Structure, and Component of PVDF-Based NFMs

**Figure 1:**
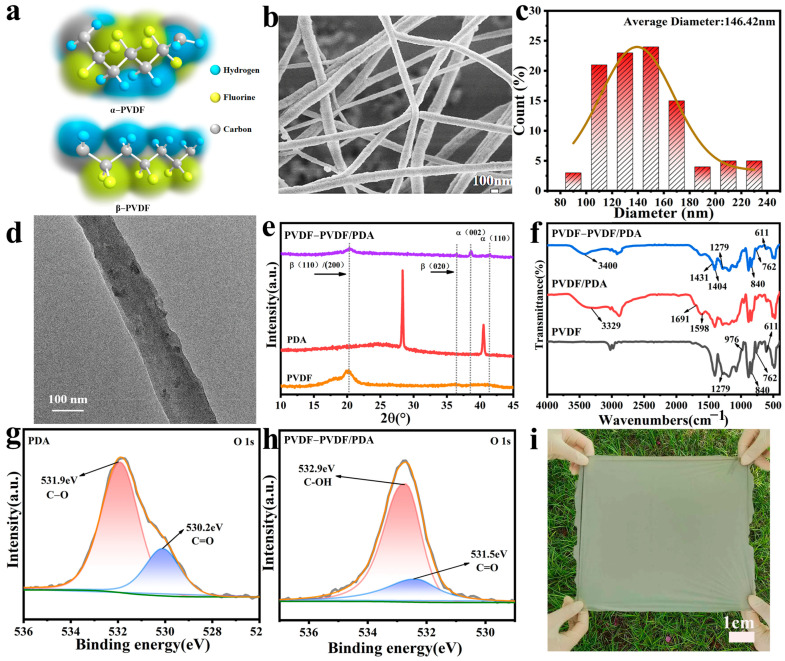
(**a**) Molecular structures of α-phase and β-phase PVDF. (**b**) SEM image and (**c**) nanofiber diameter distribution of S-3 NFMs. (**d**) TEM image of PVDF-PVDF/PDA NFMs. (**e**) XRD spectra and (**f**) FTIR spectra of PVDF, PVDF/PDA, and PVDF-PVDF/PDA NFMs. The O1s XPS spectra of (**g**) PDA and (**h**) PVDF-PVDF/PDA NFMs. (**i**) Photograph of PVDF-PVDF/PDA NFMs.

Figure 1a shows molecular structure models of the α and β phases of PVDF. During electrospinning, the static voltage could facilitate high crystallization of β-phase PVDF. Each unit cell of β-PVDF contains two molecular chains. Not only are dipole moments in the unit aligned parallel, but the dipole moments of the two molecular chains are also parallel, exhibiting TTTT transformation conformation, which enhances the polarity of the β phase. The average particle size of PDA was 55.3 nm (Figure S1a,b). The surface of PDA was rough with a rich sense of granularity (Figure S1c). PVDF nanofibers were randomly distributed and relatively straight, and the fiber surface had a large number of raised rough structures (Figure S2). All nanofibers from double-nozzle electrospun PVDF/PDA NFMs were randomly distributed but more curved, with a bumpy fiber surface, possibly due to PDA adhering to both the surface and interior of the fiber (Figure 1b,c and Figure S3). PDA can improve the dispersion and uniformity of the material and may affect the internal structure and orientation of the fiber. Figure 1d clearly shows that PDA particles were dispersed on the surface and within the fibers. The average fiber diameter of PVDF-PVDF/PDA NFMs increased with increasing PDA doping levels (Figure S3). This may be because more PDA provides more abundant adsorption sites, increasing the surface area and enhancing the roughness of the surface, which strengthens the interaction with the polymer matrix (Table 1). With increasing PDA doping amount, the rich amino and polar role of PDA makes it adhere to the interior of the fiber, resulting in a reduced mean diameter of PVDF/PDA (Figure S4). When the PDA content was 0.1 g or 0.2 g, a large number of PDA particles attached to the fiber surface, and PDA agglomeration was observed on the fiber surface (Figure S4a,d). This may be due to double-nozzle electrospinning, where the jet is influenced by external electric field effects and Coulomb forces from other jets, resulting in a small fiber diameter. At the same PDA doping amount, the diameter of nanofibers prepared by double-nozzle electrospinning was smaller than that of single-nozzle electrospinning. This may be attributed to the interaction between abundant amino groups on the PDA surface and β-PVDF in double-nozzle electrospinning [17].

XRD spectra of PDA, PVDF NFMs, and PVDF-PVDF/PDA NFMs are shown in Figure 1e. It can be seen that both PVDF and PVDF-PVDF/PDA NFMs showed a strong diffraction peak at 20.4°, which is attributed to (110)/(200) crystal surface of β-PVDF [18]. This indicates that PDA does not affect the crystal structure and phase transformation of β-PVDF in electrospun PVDF-PVDF/PDA NFMs. In addition, PVDF-PVDF/PDA NFM had major peaks at 2θ of 38.6° and 41.5°, which correspond to the α crystalline phase with (002) and (110) reflective surfaces [19]. An additional new peak, 2θ of 36.5°, is attributed to the TTTT transformation of the β phase and the corresponding (020) reflection plane. This may be because the β phase is generated by in situ electric field polarization and mechanical stretching assisted by electrospinning. Thus, the β phase was the main crystal form of PVDF-based NFMs. Therefore, the α to β phase transition was achieved during electrospinning, and it can be further verified by FTIR spectra (Figure 1f). Sharp absorption peaks of PVDF membranes at 611, 762, and 976 cm^−1^ can be attributed to vibrational absorption of α-PVDF, as well as at 840 and 1279 cm^−1^ for β-PVDF [20]. A weak band was visible in the PVDF/PDA membrane at 1691 cm^−1^, which belonged to the stretching of PDA-derived aromatic ring [21]. There were two wide absorption bands at 1598 and 3329 cm^−1^, which may be attributed to the stretching vibration of N-H and C=C groups of PDA. FTIR spectra of PVDF-PVDF/PDA NFMs were similar to PVDF and PVDF/PDA NFMs, indicating that the addition of PDA does not affect the structure of PVDF. However, the sharp absorption peak of PVDF-PVDF/PDA NFMs at 611 and 762 cm^−1^ can be attributed to the vibration absorption of α-PVDF, and at 840, 1279, 1404, and 1431 cm^−1^ to the vibration absorption of β-PVDF [22]. Moreover, the wide absorption band at 3400 cm^−1^ corresponds to the tensile vibration of PDA. The proportion of the β phase can be calculated by the following Formula (2) [23]:

(2) $$F(\beta )=\frac{A_{\beta }}{A_{\beta }+1.26A_{\alpha }}$$

where *F(β)* is the proportion of the β phase, and *A_α_* and *A_β_* are absorption peak intensities of α and β phases. According to *A_α_* and *A_β_* absorption peaks of PVDF-PVDF/PDA NFMs at 611 cm^−1^ and 1431 cm^−1^, it has been calculated that the β-phase proportion was 65.02%. This is because electrospinning can promote the transition of the nonpolar α phase to the polar β phase. The reduction of the α-phase absorption peak and significant increase of the β phase in PVDF-PVDF/PDA NFMs once again verified that PDA addition and electrospinning can promote the transition of the nonpolar α phase to the polar β phase. This result is consistent with the XRD spectrum analysis shown in Figure 1e. The surface properties and chemical structure of the membrane were further analyzed by XPS. The signal of O1s PDA demonstrated the presence of C-O and C=O (Figure 1g). O1s of PVDF-PVDF/PDA NFMs had two major peaks, and spectral peaks at 531.5 and 532.9 eV were attributed to quinine C=O and catechol C-OH (Figure 1h), respectively [24]. PVDF-PVDF/PDA NFMs still have N and F elements (Figure S5a). The canonical N1s signal corresponded to species of N in PDA. In the N1s spectrum of PDA, -N= (397.9 eV) and -NH- (399 eV) correspond to cyclic nitrogen [25] (Figure S5b). In the F1s spectrum of PVDF-PVDF/PDA, there were 688.4 eV and 688.5 eV peaks at the high binding energy (Figure S5c). Both PDA and PVDF-PVDF/PDA had similar characteristic peaks, and the results confirmed the successful construction of PDA in composite nanomaterials. Figure 1i shows the prepared large-area PVDF-PVDF/PDA NFMs, which were gray.

### 3.2. Electrical Performance of PVDF-Based NFMs

Due to the high-voltage electric field and mechanical stretching in the electrospinning process, the PVDF jet is polarized and stretched. This promotes dipole moment-oriented transition of the PVDF molecular chain from α-PVDF to β-PVDF and enhances the crystallization of β-PVDF [26]. The charge can be stored in nanofibers when electrospinning [27]. PDA is an organic polymer with a large molecular weight and strong polarity. Based on this, PVDF-PVDF/PDA NFMs have good electrical properties, which can enable PVDF-PVDF/PDA NFMs to achieve active adsorption and stable adhesion to particles by their own electrostatic force [28].

**Figure 2:**
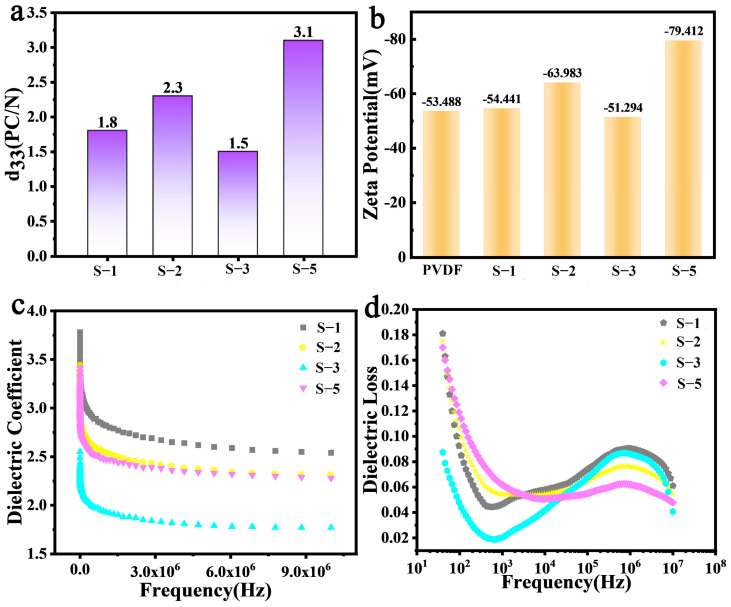
(**a**) d_33_, (**b**) Zeta potential on the solid surface, (**c**) dielectric coefficient, and (**d**) dielectric loss of S-1, S-2, S-3, and S-5 NFMs.

Figure 2 shows changes in piezoelectric coefficient (d_33_), surface potential, dielectric constant, and dielectric loss of PVDF-PVDF/PDA NFMs. D_33_ value of S-3 was 1.5 PC/N, and that of S-5 was 3.1 PC/N (Figure 2a), which indicated the piezo effect of S-5 can produce a greater charge and higher sensitivity [29]. Surface Zeta potential was a representation of the number of charges on the surface of a material, and negative values indicated that PVDF-PVDF/PDA NFMs were negatively charged [30] (Figure 2b). It can be seen that the absolute value of S-5 was the largest, at 79.412 mV. The absolute value of the surface Zeta potential of S-3 NFMs was higher than that of PVDF/PDA-3 (Figure S6). This indicated that the higher the absolute value of S-3 was, the greater its electrostatic repulsion and the more stable the system would be. Importantly, the absolute value of the surface Zeta potential of all membranes was greater than 50 mV, indicating that all membranes we prepared had relatively good stability [31]. The dielectric constant and dielectric loss of PVDF-PVDF/PDA NFMs are shown in Figure 2c,d with the increase of PDA content. A study shows that the dielectric constant and dielectric loss values of ideal matter were small [32]. The dielectric constant of S-3 decreased from 2.55 to 1.77, and the minimum dielectric loss was 0.0407. This indicated that S-3 had a small polarization charge, small response capacity, and small loss. The electrical properties of composite nanofiber membranes are realized by the interaction between functional groups on PDA and specific groups in the PVDF segment. Specifically, there are a large number of -NH- groups in composite nanostructure, and -CF_2_ in the PVDF segment forms hydrogen bonds, which can also promote the β phase transition. The charge of nanofiber membrane was mainly derived from β-PVDF. Therefore, polarized PVDF-PVDF/PDA NFMs could absorb and adhere by electrostatic effect, achieving efficient air filtration efficiency and oil–water separation [33]. It can be seen that membrane still maintains good or even higher electrical performance after filtration (Figure S6b,c). This is because the adsorption of PMs on the membrane increases surface roughness, which makes it easier to intensify friction and generate more static electricity.

### 3.3. Air Filtration Performance and Oil–Water Separation of PVDF-Based NFMs

**Figure 3:**
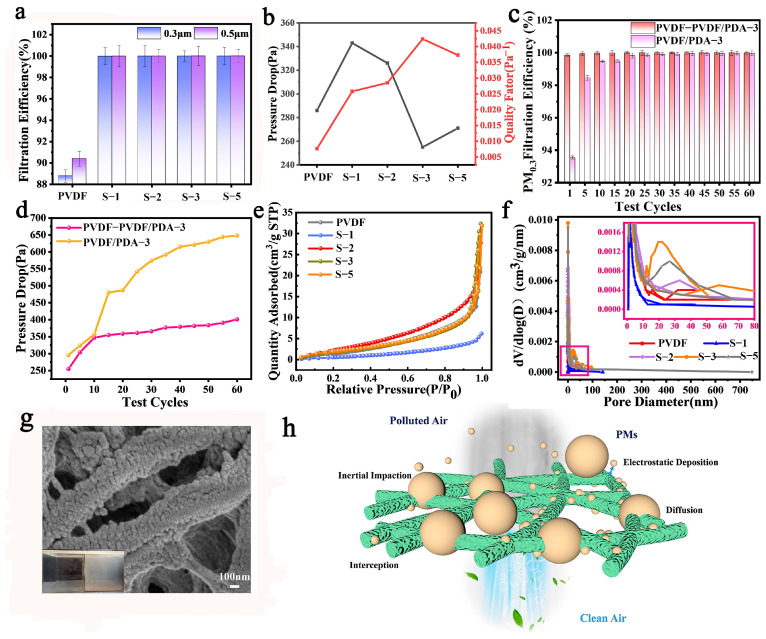
(**a**) Filtration efficiency, (**b**) pressure drop, and quality factor of PVDF, S-1, S-2, S-3, and S-5 NFMs. (**c**) Filtration efficiency and (**d**) pressure drop of S-3 and PVDF/PDA-3 under 60 cycles of filtration. (**e**) N_2_ adsorption–desorption isotherms and (**f**) pore diameter distributions of PVDF, S-1, S-2, S-3, and S-5. (**g**) SEM image of PVDF-PVDF/PDA after filtration (inset showing the filter simulation device). (**h**) Schematic of the filtration mechanism of PVDF-PDF/PDA NFMs.

Figure 3a,b shows the PM filtration properties of nanofiber membranes at the same base weight and airflow velocity. The filtration efficiency of PM_0.3_ of PVDF NFMs reached 88.799%, but the filtration efficiency of the nanofiber membrane after the addition of PDA was greatly improved. PM_0.3_ filtration efficiency of S-1 NFMs was 99.986%, but the pressure drop was 343 Pa. In particular, the PM_0.3_ filtration efficiency of S-3 NFM was as high as 99.998%, and the pressure drop was 255 Pa. The quality factor was 0.0424 Pa^−1^. This may be due to the small pore size between fibers caused by a fine fiber diameter and a bumpy surface structure, which increased the probability of PM capture, enhanced filtration efficiency, and pressure drop. Furthermore, it can also be seen that the filtration performance of the double-nozzle electrospinning nanofiber membrane was better than that of the single-nozzle electrospinning nanofiber membrane (Figure S7a,b). PM_0.3_ filtration efficiency of PVDF/PDA-3 NFMs was 99.987%, and pressure drop and quality factor were 296 Pa and 0.0351 Pa^−1^, respectively. This may be because PDA contains abundant π electron clouds, which can produce π–π interactions with other molecules in a π-containing system, thus adsorbing small molecules [34]. To better demonstrate the excellent filtration performance of the nanofiber membrane, PMs filtration efficiency of PVDF-PVDF/PDA-3 NFM and PVDF/PDA-3 NFM at different base weights and different airflow velocities were characterized (Figure S7c–f). At a basis weight of 8.82 g/m^2^ for S-3, PM_0.3_ filtration efficiency was 99.967%, and pressure drop was 144 Pa. S-3 and PVDF/PDA-3 NFMs were characterized for 60 cycles at a basis weight of 29.39 g/m^2^ and airflow velocity of 32 L/min (Figure 3c,d). The filter performance tester can emit 60,000 PM particles in one test. The nanofiber membrane was tested for filtration performance once, which means a cycle, followed by the next test without cleaning. S-3 and PVDF/PDA-3 NFMs had increasing filtration efficiency and pressure drop at 60 cycles. PM_0.3_ filtration efficiency of S-3 was 100%, which was greater than that of PVDF/PDA-3 (99.971%), but the pressure drop of 401 Pa was less than that of PVDF/PDA-3 (648 Pa). Necessarily, the filtration efficiency of S-3 NFMs had reached 99.998% at 20 times (Figure 3c,d). In addition, PDA carbon material has good adhesion, which can enhance the adsorption capacity of PMs, while PVDF provides mechanical strength and chemical stability. Therefore, the composite nanofiber membrane can not only ensure a good adsorption effect of PMs but also maintain the structural stability of the membrane, so as to ensure that the cycle neutral energy of PM adsorption does not decrease after many times. Moreover, electrospinning can produce nanofiber membranes with continuous fibers and moderate porosity, which can improve the adsorption performance and stability of the membrane and make it able to withstand more adsorption cycles.

N_2_ adsorption–desorption isotherms of PVDF, PVDF/PDA, and PVDF-PVDF/PDA NFMs all slowly increased with increasing relative pressure (Figure 3e,f), indicating that the adsorption capacity of membranes to N_2_ was weak [35]. Specific surface area and pore volume of nanofiber membranes are shown in Table 1 and Table S1. The distribution of PVDF and PVDF-PVDF/PDA NFMs was relatively consistent, with pores concentrated at 0–50 nm and belonging to mesoporous. However, the pore distribution of PVDF-PVDF/PDA NFMs was much greater than that of PVDF/PDA NFMs (Figure S8). Specific surface area (11.484 m^2^/g), microspore volume (0.003 cm^3^/g), and total pore volume (0.043 cm^3^/g) of S-3 NFM were greatly increased (Table 1) and were worse than PVDF/PDA-3, which is conducive to the adsorption of small particles. Generally speaking, the smaller the pore volume of the membrane is, the higher the filtration efficiency is, as a smaller pore volume can capture more PMs. Therefore, S-3 has better capture capability.

To more clearly illustrate the excellent filtration performance of nanofiber membranes, we performed real simulation experiments as shown in Figure 3g. The inset shows a simulated experimental setup. The cuboid was divided into two parts, with the filtered photograph on the left and the one before filtering on the right [36]. We placed the membrane at the middle junction on the left and right sides, and the pollution source was a lit cigarette. From the SEM image in Figure 3g, it was also obvious that filtered fibers adhered to many PMs of different sizes. Figure 3h more clearly indicates the filtration mechanism of nanofiber membranes. PVDF-PVDF/PDA NFM has five PM capture mechanisms, namely, the interception effect, diffusion effect, inertia effect, gravity effect, and electrostatic effect [37]. The nanofiber membranes with a bumpy structure not only had a larger specific surface area but also increased collision between fibers and PMs, leading to physical trapping, thus increasing filtration efficiency, especially for small particles. In detail, first, PVDF-PVDF/PDA NFMs have abundant adsorption sites and high specific surface area, increasing collision and adhesion between fibers and PMs and improving physical interception capability [38]. Next, strong polarity and abundant amino groups of PDA increase the active adsorption between fibers and PMs, thus producing a strong adhesion to the surface of the fiber [39]. Then, PDA has amino groups, and the hybridization mode of their N atoms is sp^3^ hybridization, with a pair of lone pair electrons [40]. The electronegativity of the F atom in PVDF is stronger than that of N, which will attract electrons to the N atom and generate electrostatic attraction, thereby improving the electrostatic effect of the nanofiber membrane. Finally, high voltage electric field and mechanical stretching of electrospinning promote high crystallization of β-PVDF, and electrostatic action of surface charge generated by β-PVDF adsorbs PMs in the atmosphere [41]. Table S2 compares the comprehensive properties of various PVDF-based nanofiber membranes. It can be seen that compared with other PVDF-based nanofiber membranes, our work has finer nanofiber diameter, higher PM filtration efficiency, and good electrical performance, which can be used in high-efficiency PM capture.

**Figure 4:**
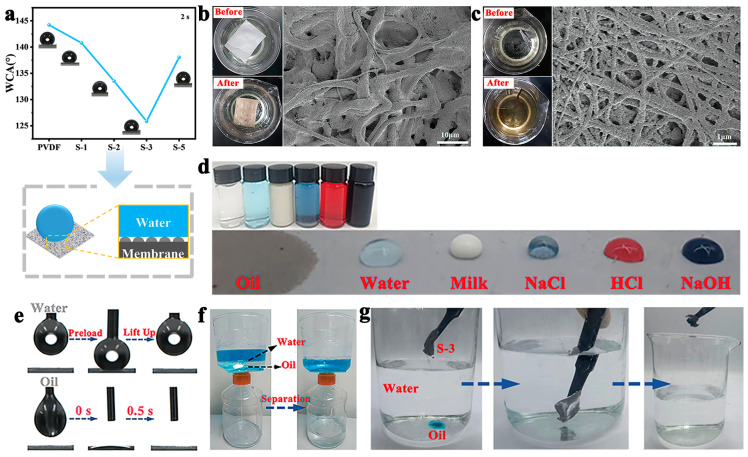
(**a**) WCA of PVDF and PVDF-PVDF/PDA NFMs. (**b**,**c**) Photos (top: before testing, bottom: after testing) and SEM images of PVDF and PVDF-PVDF/PDA-3 NFMs after testing in NaCl (0.5 mol/L) solution. (**d**) Photos of PVDF-PVDF/PDA NFMs surface with oil, water, milk, NaCl, HCl, and NaOH droplets. (**e**) Dynamic photos showing water and oil behavior on PVDF-PVDF/PDA membrane. (**f**) Separation process of oil–water mixture (blue liquid represents water). (**g**) Photo of PVDF-PVDF/PDA membrane with adsorbed oil in water (blue droplet represents oil).

The wettability of the membrane plays an important role in its permeability and antifouling properties. WCA is an important method to characterize the wetting properties of the membrane surface, so we systematically studied the wetting behavior of the nanofiber membrane. Figure 4a, S9 shows the WCA of PVDF, PVDF-PVDF/PDA, and PVDF/PDA. PVDF membrane was a hydrophobic membrane (WCA was 144.217°). Hydrophobic PVDF nanofiber membrane shows that it has a certain repelling effect on water but has a relatively good affinity for oil substances, which provides a basic physical property difference for oil–water separation to a certain extent. After the addition of PDA, WCA decreased, which was due to the nanofiber membrane with many hydrophilic groups such as hydroxyl and amino groups [42]. At the same amount of PDA doping amounts, the water contact angle of PVDF/PDA (Figure S9a) was smaller than that of the double-nozzle electrospinning membrane, which also indicated that PVDF is hydrophobic, and WCA decreased under the influence of the hydrophilic group of PDA [43]. In addition, with an increase in PDA, the water contact angle showed a trend of decreasing first, and then rising. WCA of S-3 reached its minimum, which was 125.863°. This may be due to the effects of the morphological change and PVDF on the surface properties. More importantly, there were tiny protrusions on the surface of the nanofibers, which form an extremely thin layer of air between protrusions, preventing water droplets from infiltrating the fiber surface and automatically aggregating to form water droplets (as shown in the inset of Figure 4a), thus demonstrating hydrophobicity. To illustrate the possibility of prolonged use of the filter membrane, S-3 NFMs were characterized. As can be seen, the WCA of S-3 NFM showed a downward trend within 30 min (water contact angle was 91.429°) (Figure S9b), but it was still a hydrophobic membrane. This indicated that the nanofiber membrane was not easy to wet and could be used for a long time. Necessarily, the decrease in WCA contributes to the dispersion and adsorption process of membranes in water.

PVDF and PVDF-PVDF/PDA NFMs were put into 0.5 mol/L NaCl solution for two weeks, as shown in Figure 4b,c. When PVDF NFMs were in solution, the structure of the fiber was destroyed. However, PVDF-PVDF/PDA NFMs can maintain good fiber structure, and a large number of particles were adsorbed on the surface of the fiber. This may be because the specific surface area and other microstructures of the composite nanofiber membrane provide a basis for its adsorption in water. Moreover, modification of hydrophilic groups of PDA was conducive to the adsorption of membranes in water. Therefore, the composite nanofiber membrane can maintain good adsorption properties and fiber structure in a wet environment for a long time. This can provide ideas for its application in masks, protective clothing, and other fields. PVDF-PVDF/PDA nanofiber membrane was extremely lipophilic and hydrophobic to water, milk, saturated salt water (NaCl), acid solution (HCl, PH = 1), and alkaline solution (NaOH, PH = 13) (Figure 4d). PVDF-PVDF/PDA membrane was very good for oil infiltration and has a high contact angle for saturated salt water, acid solution, and alkaline solution. These characteristics make oil substances easier to spread on the surface of the nanofiber membrane. We studied the infiltration of the fibrous membrane by oil and water using a water contact angle tester. We used dichloromethane as the oily material. It can be seen that the water droplets completely leave the membrane surface after contact with the membrane, and the oil quickly diffuses on the membrane surface, indicating the good hydrophobic and lipophilic of the fibrous membrane (Figure 4e). PVDF-PVDF/PDA nanofiber membranes were composed of nanofibers with small fiber diameters and interconnected pores. This microstructure makes membranes have high porosity, can provide a larger specific surface area, and increases the contact area with the oil–water mixture, which is conducive to oil–water separation. Then, the ability of the PVDF-PVDF/PDA membrane to separate the oil–water mixture was investigated. It can be seen that transparent oil can easily penetrate the fibrous membrane, which is collected in the bottle below, and the blue aqueous solution remains above the container (Figure 4f). PVDF-PVDF/PDA membrane adsorbs methylene chloride (blue) in the water (Figure 4g), also indicating the hydrophobic oil affinity of the membrane. Due to the hydrophilicity of PDA carbon material and the hydrophobicity of PVDF, adhesion between water and the membrane surface is strong, while adhesion between oil droplets and the membrane surface is weak. In this case, oil droplets are more likely to be pushed by the water phase or taken away by water flow at the interface. Therefore, the synergistic action of surface charge and hydrophobicity of composite nanofiber membranes causes small oily droplets to aggregate and separate from trapped miscibility. This indicates that the composite nanofiber membrane has great potential in the field of oil–water separation.

### 3.4. Acid and Alkali Resistance, Mechanical Property, Thermal Stability, and Flame Resistance of PVDF-Based NFMs

**Figure 5:**
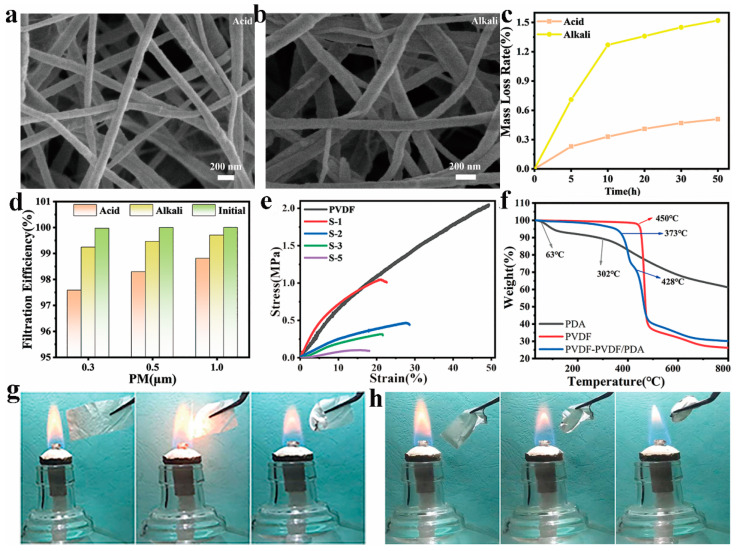
SEM images of PVDF-PVDF/PDA NFMs after a 5 h immersion in (**a**) HCl and (**b**) NaOH solution. (**c**,**d**) Mass loss after varying immersion time in HCl and NaOH solution and filtration efficiency after 5 h treatment. (**e**) Stress-strain curves of PVDF, S-1, S-2, S-3, and S-5. (**f**) TGA curves of PDA, PVDF, and PVDF-PVDF/PDA NFMs. Photographs showing (**g**) PVDF and (**h**) PVDF-PVDF/PDA NFMs under combustion.

Resistance to chemical erosion is of great importance for medical workers and in the field of water and oil separation. After soaking PVDF-PVDF/PDA nanofiber membrane in an acidic solution (HCl, PH = 1) and alkaline solution (NaOH, PH = 15) for 5 h, fiber morphology was unchanged (Figure 5a,b). After 50 h of chemical erosion, the weight of the fiber film was slightly lost (Figure 5c), and filtration efficiency was also slightly reduced (Figure 5c) after 5 h treatment, but the filtration efficiency of PM_0.3_ was still greater than 97.5%. This is because the molecular chain of PVDF contains a large number of fluorine atoms, and this structure gives the membrane excellent chemical stability and corrosion resistance to erosion of most acids, bases, and organic solvents. It thus indicates that composite nanofibrous membranes are resistant to acid and base erosion. Figure 5e is a characterization of the mechanical properties of PVDF-PVDF/PDA NFMs. PVDF NFM showed excellent mechanical properties with stress and strain of 2.052 MPa and 49.382%, but mechanical properties decreased due to the addition of PDA. The stress showed a decreasing trend with increasing PDA content. PVDF/PDA NFMs showed the same trend. Moreover, at the same PDA content, PVDF-PVDF/PDA NFMs had less stress and strain than PVDF/PDA NFMs (Figure S10), which may be because two jets repel each other due to Coulomb repulsion in double-nozzle electrospinning [44].

The TGA curves of PDA, PVDF powder, and PVDF-PVDF/PDA NFMs are shown in Figure 5f. PDA degraded at 63 °C, probably due to evaporation of residual water in PDA. The maximum weight loss peak was observed at 302 °C. PVDF powder showed a maximum degradation rate of 450 °C. For PVDF-PVDF/PDA NFMs, there were two thermal degradation phases. Thermal degradation occurred at 373 °C, probably because of the degradation of PDA, While the 428 °C degradation occurred due to the thermal degradation of the PVDF matrix. The final residual rate of the nanofiber membrane was 30.103%. Results show that PDA can promote the thermal decomposition of nanofiber membranes. Figure 5g,h shows the flame resistance properties of PVDF and PVDF-PVDF/PDA-3 NFMs [45]. The membrane was tailored into a 2–3 cm rectangle. We used a lit alcohol lamp as a source of fire. The PVDF nanofiber membrane was extinguished and retained its original form immediately after leaving the fire source. PVDF-PVDF/PDA NFMs automatically curled up when approaching the fire source and were extinguished immediately after leaving the fire source in 3 s, but they still maintained the curled appearance. On the one hand, because F atoms release a large amount of fluorine gas during combustion, they help suppress flames. On the other hand, the inert gas released from the decomposition of the -NH group in PDA dilutes the flame flow in the gas phase. Therefore, the membrane had some self-quenching properties [46,47]. Table S2 compares the comprehensive properties of various PVDF-based nanofiber membranes. By comparing diameter, *η*, Δ*P*, *QF*, electrical performance, and flame resistance with other filtration materials, our work has more outstanding efficiency in PM capture and oil–water separation, especially in the field of personal protection in fire and other emergencies and household purification.

## 4. Conclusions

In summary, our research focused on the successful fabrication of PVDF-PVDF/PDA NFMs via double-nozzle electrospinning. During the electrospinning process, a precisely controlled high-voltage electric field was applied, which not only facilitated the formation of nanofibers but also influenced their morphological and structural characteristics. The incorporation of PDA into the PVDF matrix was a key aspect of our study. PDA, with its rich surface groups, played a crucial role in enhancing the properties of nanofiber membranes. β-PVDF phase, induced by electrospinning conditions, further contributed to excellent filtration and separation capabilities of membranes. When examining the physical characteristics of membranes, we found that PVDF-PVDF/PDA-3 NFM, in particular, had a diameter of 146.42 nm. In terms of filtration performance, the membrane demonstrated an outstanding PM_0.3_ filtration efficiency of 99.967%. This efficiency was measured using a customized air filtration test rig, where a controlled flow of air containing PM_0.3_ particles was passed through the membrane. A low pressure drop of 144 Pa was simultaneously recorded, indicating the membrane’s ability to maintain efficient filtration with minimal energy consumption. The filter membrane also demonstrated excellent hydrophobicity and lipophilicity. Oil–water separation experiments were also carried out, where the membrane effectively separated oil from water mixtures, highlighting its high-performance capabilities in this area. Additionally, the composite nanofiber membrane displayed strong acid and alkali resistance. Moreover, PVDF-PVDF/PDA NFMs had excellent flame retardancy, self-extinguishing within 3 s, ensuring suitability for protective clothing applications. In conclusion, our strategy of fabricating PVDF-PVDF/PDA NFMs provides valuable insights for the development of multifunctional fiber membrane materials. The detailed understanding of the relationship between the fabrication process, material composition, and resulting properties can serve as a foundation for further research and optimization of similar membrane materials.

## Figures and Tables

**Table 1:** Specific surface area, pore volume, and microspore volume of PVDF-PVDF/PDA NFMs with different PDA doping levels.

Samples	Surface Area (m^2^/g)	Pore Volume (cm^3^/g)	Microspore Volume (cm^3^/g)
PVDF	7.337	0.032	0.002
S-1	2.824	0.009	0.001
S-2	8.654	0.050	0.002
S-3	11.484	0.043	0.003
S-5	9.176	0.049	0.002

## Data Availability

The original contributions presented in the study are included in the article/Supplementary Material. Further inquiries can be directed to the corresponding author.

## Supplementary Materials

The following supporting information can be downloaded at: https://www.mdpi.com/article/10.3390/polym17050703/s1. Figure S1: (a,b) SEM images and diameter distribution of PDA carbon materials. (c) Pore diameter distribution of PDA carbon materials; Figure S2: (a,b) SEM image and diameter distribution of PVDF; Figure S3: SEM images and diameter distribution of (a-b) S-1, (c,d) S-2, (e,f) S-5; Figure S4: SEM images and diameter distribution of (a,b) PVDF/PDA-1, (c,d) PVDF/PDA-2, (e,f) PVDF/PDA-3, (g,h) PVDF/PDA-5.; Figure S5: (a) XPS full spectrum, and XPS spectra of (b) N1s for PDA and (c) F1s for PVDF-PVDF/PDA NFMs; Figure S6: (a) Zeta potential on the solid surface of PVDF/PDA NFMs. (b) Changes in d33 and zeta potential on the solid surface of PVDF-PVDF/PDA-3 NFMs after filtration. (c,d) Changes in dielectric coefficient and dielectric loss of PVDF-PVDF/PDA NFMs after filtration; Figure S7: (a,b) PMs filtration efficiency, pressure drop and quality factors of PVDF/PDA NFMs. PM0.3 filterability of PVDF-PVDF/PDA-3 and PVDF/PDA-3 NFMs with (c,d) different base weights and (e,f) different airflow velocities; Figure S8: Pore diameter distributions of PVDF/PDA NFMs; Figure S9: (a) Water contact angle of PVDF/PDA-x NFMs. (b) Water contact angle changes with time of S-3; Figure S10: Stress-strain curves of PVDF/PDA-x NFMs; Table S1: Specific surface area, total pore volume and microspore volume of PVDF/PDA NFMs with different PDA doping amounts; Table S2: Nanofiber membrane various performance comparison from the reported literature. References [1,23,31,48,49,50,51,52,53,54,55,56,57,58] are cited in the Supplementary Materials.

## References

[1] Sanyal A., Sinha-Ray S. (2021). Ultrafine PVDF nanofibers for filtration of air-borne particulate matters: A comprehensive review. Polymers.

[2] Chen J., Guo C., Zhang Q., Wu X., Zhong L., Zheng Y. (2023). Preparation of transparent, amphiphobic and recyclable electrospun window screen air filter for high-efficiency particulate matters capture. J. Membr. Sci..

[3] Liang G., Zhang W., Zhang X., Yu J., Zhang S., Ding B. (2024). Ultralight, superelastic, and antibacterial micro/nanofibrous sponges with dual-network interwoven structure for warmth retention. Compos. Commun..

[4] Li P., Jiang D., Xu C., Su Z. (2025). A scanning manometry method to image the atomization pressure field for pre-debugging the nanocomposite spraying system. Compos. Commun..

[5] Zhou N., Gao Y., Huo Y., Zhang K., Zhu J., Chen M., Zhu L., Dong Y., Gao H., Soo Kim I. (2023). Biodegradable micro-nanofiber medical tape with antibacterial and unidirectional moisture permeability. Chem. Eng. J..

[6] Ren X., Liu H., Wang J., Yu J. (2024). Electrospinning-Derived functional carbon-based materials for energy conversion and storage. Chin. Chem. Lett..

[7] Liu Y., Wang L., Liu Y., Zhang F., Leng J. (2024). Recent progress in shape memory polymer composites: Driving modes, forming technologies, and applications. Compos. Commun..

[8] Fan C., Long Z., Zhang Y., Mensah A., He H., Wei Q., Lv P. (2023). Robust integration of energy harvesting with daytime radiative cooling enables wearing thermal comfort self-powered electronic devices. Nano Energy.

[9] Wang C., He X., Zhu G., Li X., Zhu X., Chen R., Tian S., Li X., Zhu J., Shao J. (2024). Extreme orientation of stereocomplexed poly (lactic acid) induced ultrafine electroactive nanofibers for respiratory healthcare and intelligent diagnosis. ACS Sustain. Chem. Eng..

[10] Li Y., Wang D., Xu G., Qiao L., Li Y., Gong H., Shi L., Li D., Gao M., Liu G. (2021). ZIF-8/PI Nanofibrous Membranes With High-Temperature Resistance for Highly Efficient PM_0.3_ Air Filtration and Oil-Water Separation. Front. Chem..

[11] Singh R.K., Lye S.W., Miao J. (2021). Holistic investigation of the electrospinning parameters for high percentage of β-phase in PVDF nanofibers. Polymer.

[12] Chen J., Ayranci C., Tang T. (2023). Piezoelectric performance of electrospun PVDF and PVDF composite fibers: A review and machine learning-based analysis. Mater. Today Chem..

[13] Dai G., Chu J.C.H., Chan C.K.W., Choi C.H.J., Ng D.K.P. (2021). Reactive oxygen species-responsive polydopamine nanoparticles for targeted and synergistic chemo and photodynamic anticancer therapy. Nanoscale.

[14] Zhang Y., Zeng Z., Ma X.Y.D., Zhao C., Ang J.M., Ng B.F., Wan M.P., Wong S.-C., Wang Z., Lu X. (2019). Mussel-Inspired approach to cross-linked functional 3D nanofibrous aerogels for energy-efficient filtration of ultrafine airborne particles. Appl. Surf. Sci..

[15] Ma F., Zhang N., Wei X., Yang J., Wang Y., Zhou Z. (2017). Blend-electrospun poly (vinylidene fluoride)/polydopamine membranes: Self-polymerization of dopamine and the excellent adsorption/separation abilities. J. Mater. Chem. A.

[16] Li Y., Yuan D., Geng Q., Yang X., Wu H., Xie Y., Wang L., Ning X., Ming J. (2021). MOF-Embedded bifunctional composite nanofiber membranes with a tunable hierarchical structure for high-efficiency PM_0.3_ purification and oil/water separation. ACS Appl. Mater. Interfaces.

[17] Liu Y., Liu S., Xie X., Li Z., Wang P., Lu B., Liang S., Tang Y., Zhou J. (2022). A functionalized separator enables dendrite-free Zn anode via metal-polydopamine coordination chemistry. InfoMat.

[18] Wang Z., Sahadevan R., Crandall C., Menkhaus T.J., Fong H. (2020). Hot-Pressed PAN/PVDF hybrid electrospun nanofiber membranes for ultrafiltration. J. Membr. Sci..

[19] Yang Y., Huang E., Dansawad P., Li Y., Qing Y., Lv C., Cao L., You S., Li Y., Li W. (2023). Superhydrophilic and underwater superoleophobic PVDF-PES nanofibrous membranes for highly efficient surfactant-stabilized oil-in-water emulsions separation. J. Membr. Sci..

[20] Li X., Wang C., Huang X., Zhang T., Wang X., Min M., Wang L., Huang H., Hsiao B.S. (2018). Anionic surfactant-triggered steiner geometrical poly (vinylidene fluoride) nanofiber/nanonet air filter for efficient particulate matter removal. ACS Appl. Mater. Interfaces.

[21] Zhao K., Wei S., Cao M., Wang M., Li P., Li H., Zhang X., Zhang Y., Chen Y. (2024). Dielectric polyimide composites with enhanced thermal conductivity and excellent electrical insulation properties by constructing 3D oriented heat transfer network. Compos. Sci. Technol..

[22] Wu X., Wu X., Wang T., Zhao L., Truong Y.B., Ng D., Zheng Y., Xie Z. (2020). Omniphobic surface modification of electrospun nanofiber membrane via vapor deposition for enhanced anti-wetting property in membrane distillation. J. Membr. Sci..

[23] Su C., Zhang L., Zhang Y., Huang X., Ye Y., Xia Y., Gong Z., Qin X., Liu Y., Guo S. (2023). P(VDF-TrFE)/BaTiO_3_ nanofibrous membrane with enhanced piezoelectricity for high PM_0.3_ filtration and reusable face masks. ACS Appl. Mater. Interfaces.

[24] Wang T., Wang P., Pan L., He Z., Dai L., Wang L., Liu S., Jun S.C., Lu B., Liang S. (2022). Stabling zinc metal anode with polydopamine regulation through dual effects of fast desolvation and ion confinement. Adv. Energy Mater..

[25] Wang M., Cheng X., Jiang G., Xie J., Cai W., Li J., Wang Y. (2022). Preparation and pervaporation performance of PVA membrane with biomimetic modified silica nanoparticles as coating. J. Membr. Sci..

[26] Zhang M., Tan Z., Zhang Q., Shen Y., Mao X., Wei L., Sun R., Zhou F., Liu C. (2023). Flexible self-powered friction piezoelectric sensor based on structured PVDF-based composite nanofiber membranes. ACS Appl. Mater. Interfaces.

[27] Wang C., Song X., Li T., Zhu X., Yang S., Zhu J., He X., Gao J., Xu H. (2023). Biodegradable electroactive banofibrous air filters for long-term respiratory healthcare and self-powered monitoring. ACS Appl. Mater. Interfaces.

[28] Deng H., Zhao N., You J., Pan Z., Xing B., Ye Y., Lai B., Wang Y., Lu T., Liu X. (2024). Removal of bisphenol A through peroxymonosulfate activation with N-doped graphite carbon spheres coated cobalt nanoparticles catalyst: Synergy of nonradicals. Chin. Chem. Lett..

[29] Kim S., Lee H. (2021). Piezoelectric ceramics with high d_33_ constants and their application to film speakers. Materials.

[30] Ding S., Cao Y., Huang F., Wang Y., Li J., Chen S. (2022). Spontaneous polarization induced electrostatic charge in washable electret composite fabrics for reusable air-filtering application. Compos. Sci. Technol..

[31] Chen M., Jiang J., Feng S., Low Z.-X., Zhong Z., Xing W. (2021). Graphene oxide functionalized polyvinylidene fluoride nanofibrous membranes for efficient particulate matter removal. J. Membr. Sci..

[32] Yang T., Zhu X., Zhang Y., Ke L., Zhu J., Huang R., Li S., Zhu Y., Zhang S., Zhong G.-J. (2024). Nanopatterning of beaded poly (lactic acid) nanofibers for highly electroactive, breathable, UV-shielding and antibacterial protective membranes. Int. J. Biol. Macromol..

[33] Wang M.J., Yang J., Peng L., Bai Y., Liu Z., Yang X., Lu H., Zhou B., Jiang N., He G. (2024). Optimizing the size and electronic effects of core-shell heterostructures via well-constructed Ru clusters encapsulated in N-doped carbon layers. Chin. Chem. Lett..

[34] Liu S., Zhang C., Zhou Y., Zhang F., Duan X., Liu Y., Zhao X., Liu J., Shuai X., Wang J. (2023). MRI-Visible mesoporous polydopamine nanoparticles with enhanced antioxidant capacity for osteoarthritis therapy. Biomaterials.

[35] Cheng N., Miao D., Wang C., Lin Y., Babar A.A., Wang X., Wang Z., Yu J., Ding B. (2023). Nanosphere-Structured hierarchically porous PVDF-HFP fabric for passive daytime radiative cooling via one-step water vapor-induced phase separation. Chem. Eng. J..

[36] Liu H., Zhang S., Liu L., Yu J., Ding B. (2019). A fluffy dual-network structured nanofiber/net filter enables high-efficiency air fltration. Adv. Funct. Mater..

[37] Zhu G., Li X., Li X.-P., Wang A., Li T., Zhu X., Tang D., Zhu J., He X., Li H. (2023). Nanopatterned electroactive polylactic acid nanofibrous MOFilters for efficient PM_0.3_ filtration and bacterial inhibition. ACS Appl. Mater. Interfaces.

[38] Zong D., Bai W., Geng M., Yin X., Wang F., Yu J., Zhang S., Ding B. (2023). Direct synthesis of elastic and stretchable hierarchical structured fiber and graphene-based sponges for noise reduction. ACS Nano.

[39] Peng L., Hung C.-T., Wang S., Zhang X., Zhu X., Zhao Z., Wang C., Tang Y., Li W., Zhao D. (2019). Versatile nanoemulsion assembly approach to synthesize functional mesoporous carbon nanospheres with tunable pore sizes and architectures. J. Am. Chem. Soc..

[40] Liang C., Li J., Chen Y., Ke L., Zhu J., Zheng L., Li X.-P., Zhang S., Li H., Zhong G.-J. (2023). Self-charging, breathable, and antibacterial poly (lactic acid) nanofibrous air filters by surface engineering of ultrasmall electroactive nanohybrids. ACS Appl. Mater. Interfaces.

[41] Li J., Yin J., Wee M.G.V., Chinnappan A., Ramakrishna S. (2023). A self-Powered piezoelectric nanofibrous membrane as wearable tactile sensor for human body motion monitoring and recognition. Adv. Fiber Mater..

[42] Zhu Y., Yan J., Liu J., Chen H., Gui J., Wu C., Zhu X., Yin P., Liu M., Zhang Y. (2022). Multi-mimic activities of Co_3_O_4_ nanopolyhedrons and application in regulating the content of intracellular hydrogen peroxide/oxygen. ACS Appl. Nano Mater..

[43] Dong W., Zhao Z., Liu F., Li P., Wang L., Zhou Y., Shen Y., Lang C., Deng B., Li H. (2023). PVDF nanofiber modified with ZnO nanowires/polydopamine for the treatment of sewage containing heavy metals, organic dyes, and bacteria. ACS Appl. Mater. Interfaces.

[44] Zhi Q., Li D., Zhang Z., Fu L., Zhu W. (2023). High-Content continuous carbon fiber reinforced multifunctional prepreg filaments suitable for direct 3D-printing. Compos. Commun..

[45] Xue M., Qin R., Peng C., Xia L., Xu Y., Luo W., Chen G., Zeng B., Liu X., Dai L. (2024). *o*-Vanillin based MOFs as phosphorus-free flame retardant for reinforced epoxy resin. Compos. Commun..

[46] Si Y., Yang J., Wang D., Shi S., Zhi C., Huang K., Hu J. (2023). Bioinspired hierarchical multi-protective membrane for extreme environments via co-electrospinning-electrospray strategy. Small.

[47] Wang H., Wang Z., Shi Y., Liu M., Yao A., Feng Y., Fu L., Lv Y., Yang F., Yu B. (2023). Supramolecular engineered ultrathin MXene towards fire safe polylactic acid composites. Compos. Commun..

[48] Peng Z., Shi J., Xiao X., Hong Y., Li X., Zhang W., Cheng Y., Wang Z., Li W.J., Chen J. (2022). Self-charging electrostatic face masks leveraging triboelectrification for prolonged air filtration. Nat. Commun..

[49] Al-Attabi R., She F., Zhao S., Dumée L.F., Schütz J.A., Xing W., Zhong Z., Kong L. (2023). Durable and comfortable electrospun nanofiber membranes for face mask applications. Sep. Purif. Technol..

[50] Wang H., Bao Y., Yang X., Lan X., Guo J., Pan Y., Huang W., Tang L., Luo Z., Zhou B. (2022). Study on filtration performance of PVDF/PUL composite air filtration membrane based on far-field electrospinning. Polymers.

[51] Bui T.T., Shin M.K., Jee S.Y., Long D.X., Hong J., Kim M.-G. (2022). Ferroelectric PVDF nanofiber membrane for high-efficiency PM_0.3_ air filtration with low air flow resistance. Colloids Surf. A.

[52] Zheng J., Zhou X., Wang B., Dai F., Liu J. (2024). Modified PVDF/PMMA/SiO_2_ composite nanofibrous membrane in airborne filtration: Transparency, mechanical properties and filtration performance. J. Environ. Chem. Eng..

[53] Moon J., Bui T.T., Jang S., Ji S., Park J.T., Kim M.-G. (2021). A highly efficient nanofibrous air filter membrane fabricated using electrospun amphiphilic PVDF-g-POEM double comb copolymer. Sep. Purif. Technol..

[54] Gao H., Li Z.-J., Xu X.-F., Wang N., Yang M.-Y., Long Y.-Z., Zhang H.-D. (2024). Electrospinning dual energy-saving design of PVDF-HFP nanofiber films for passive radiant cooling and air filtration. AIP Adv..

[55] Wu Y., Li X., Zhong Q., Wang F., Yang B. (2023). Preparation and filtration performance of antibacterial PVDF/SiO_2_/Ag composite nanofiber membrane. J. Build. Eng..

[56] Geng Q., Dong S., Li Y., Wu H., Yang X., Ning X., Yuan D. (2022). High-performance photoinduced antimicrobial membrane toward efficient PM_2.5-0.3_ capture and oil-water separation. Sep. Purif. Technol..

[57] Toptaş A., Çalışır M.D., Kılıç A. (2023). Production of ultrafine PVDF nanofiber/nanonet-based air filters via the electroblowing technique by employing PEG as a pore-forming agent. ACS Omega.

[58] Liu F., Li M., Li F., Weng K., Qi K., Liu C., Ni Q., Tao X., Zhang J., Shao W. (2020). Preparation and properties of PVDF/Fe_3_O_4_ nanofibers with magnetic and electret effects and their application in air filtration. Macromol. Mater. Eng..

