# Supplementary material for: Bicomponent Electrospinning of PVDF-Based Nanofiber Membranes for Air Filtration and Oil–Water Separation
Source: Polymers (Basel). 2025 Mar 6;17(5):703. doi: 10.3390/polym17050703 (PMC11902515; doi:10.3390/polym17050703)
Supplement: Supplementary file 1 [file polymers-17-00703-s001.zip › polymers-3515835-supplementary.pdf]

Supporting Information

Bicomponent Electrospinning of PVDF-Based Nanofiber Membranes for Air Filtration and Oil–Water Separation

Tianxue Feng [1], Lin Fu [2], Zhimei Mu [1], Wenhui Wei [1], Wenwen Li [1], Xiu Liang [1], Liang Ma [3], Yitian Wu [1], Xiaoyu Wang [1], Tao Wu [1], Meng Gao [1], Guanchen Xu [1] and Xingshuang Zhang [1,*]

[1] Advanced Materials Institute, Qilu University of Technology (Shandong Academy of Sciences), Jinan 250014, China
[2] Sino Science and Technology Co., Ltd., Dong Ying 257000, China
[3] Guochen Industrial Group Co., Ltd., Jinan 250300, China
* Correspondence: xszhang@qlu.edu.cn

Synthesis of the PDA

PDA were prepared using carbonization-activation method. Firstly, 1.0 g of dopamine hydrochloride was dissolved in 50 mL of ultrapure water and stirred for 10 min. Then, dopamine hydrochloride aqueous solution was added into a mixture of ethanol and ammonia ($V_{Ethanol}$: $V_{Ammonia}$ = 50:1). The mixed solution was sealed and stirred for 10 h at 25°C, obtaining PDA mixed solution. PDA mixed solution was extracted with 0.12 μm microporous filter membrane. Secondly, extracted solid was dried at 50°C for 10 h to obtain PDA precursor. The mixed and grinded PDA precursor and KOH (m_{PDA}: m_{KOH}=1:1) for alkalization was annealed at 300°C for 1 h under Ar atmosphere. Thirdly, the mixture materials after annealing was washed with dilute hydrochloric acid solution (1 mol/L). Finally, extracted material was dried at 60°C for 12 h, obtaining PDA materials.

Measurement and characterization

The surface morphology and structure of nanofibers were observed by High-Resolution Transmission Electron Microscope (HRTEM, FEI Talos F200s, USA) and Scanning Electron Microscopy (SEM, JSM-7610FPlus, Japan). Chemical composition was characterized by Fourier Transform Infrared spectroscopy (FTIR) (VERTRX 70, Germany). Crystal structure was analysed using X-ray diffraction (XRD, Smartlab, Japan). Electronic states of elements were measured by X-ray photoelectron spectroscopy (XPS, Thermo ESCALAB 250Xi, USA) with a monochromatic Al Kα (1486.6 eV). Thermal stability was investigated by heating 10°C/min N_2 flow with Thermal Analyzer (TGA, METTLERTOLEDO). Water contact angle was measured using Contact Angle Measuring Instrument (SDC-100, SINDIN, China). N_2 adsorption desorption isotherm was detected using BET surface analyzer (Quantachrome Autosorb-iQ, USA), and aperture distribution was calculated by Barrett Joyner-Horenda (BJH) and Horvath-Kawazoe (HK) methods. Mechanical properties were tested using Electronic Universal Test Machine (UTM2102, China) at a loading rate of 1 mm/min. Air filtration performance was tested using G506 Particle Filtration Efficiency Tester. The Solid Surface Zeta Potential Tester (Anton Paar surpass) was used to test surface potential of membranes. Dielectric Tester was used to test membrane dielectric constant and dielectric loss. D_{33} Tester (Piezoconstant test, ZJ-3A) was used to test the d_{33} coefficient of membrane.

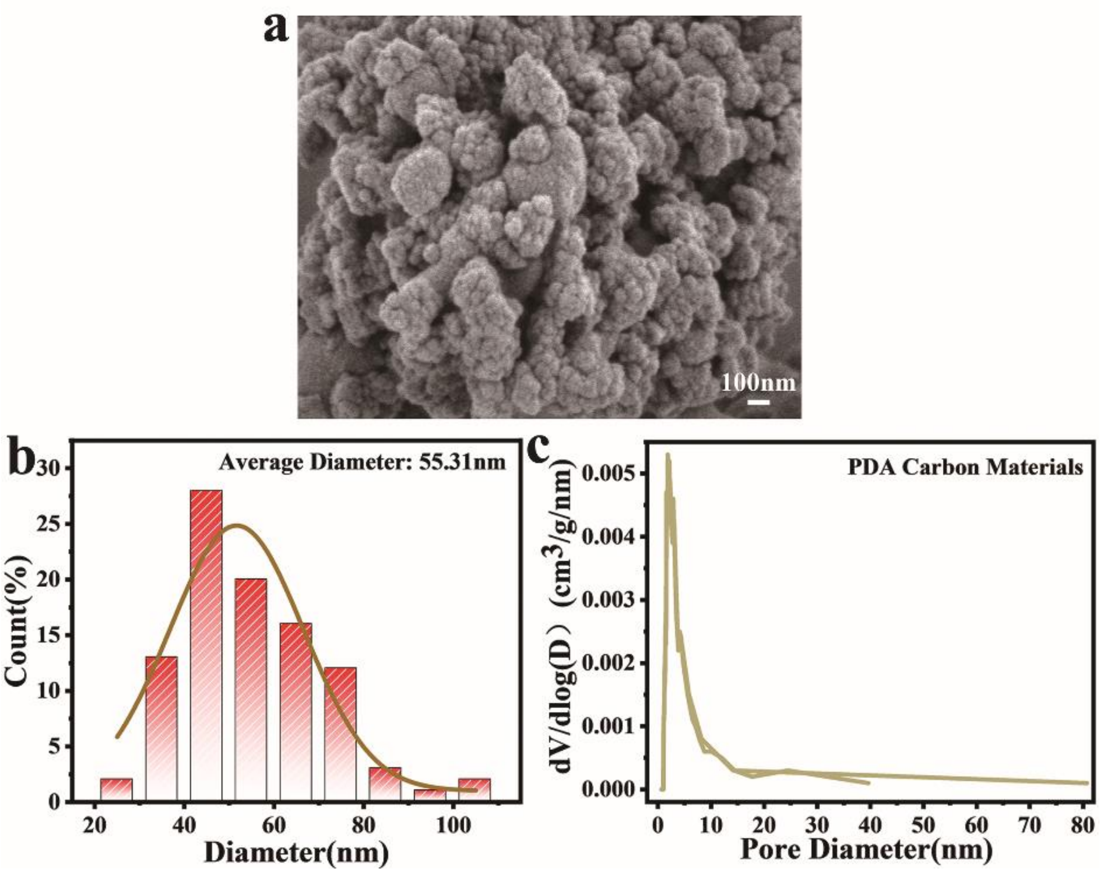

Figure S1. (a-b) SEM images and diameter distribution of PDA carbon materials. (c) Pore diameter distribution of PDA carbon materials.

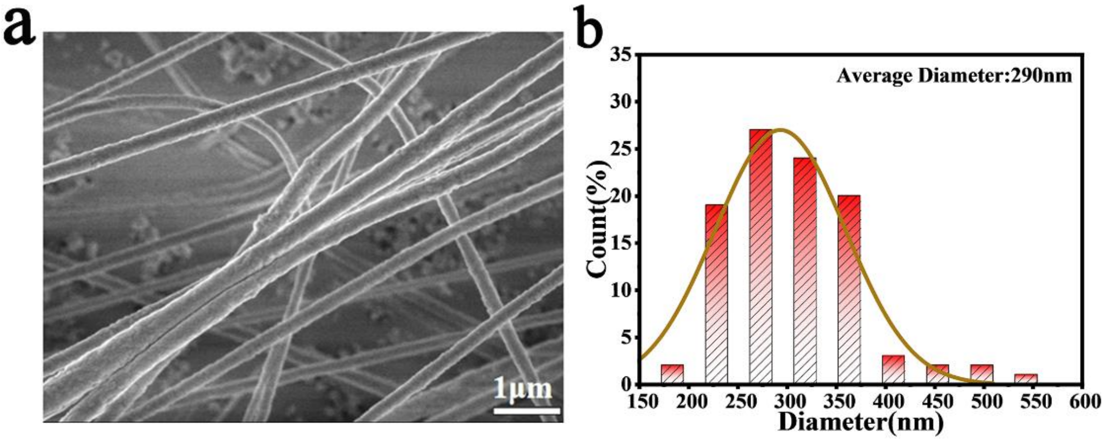

Figure S2. (a-b) SEM image and diameter distribution of PVDF.

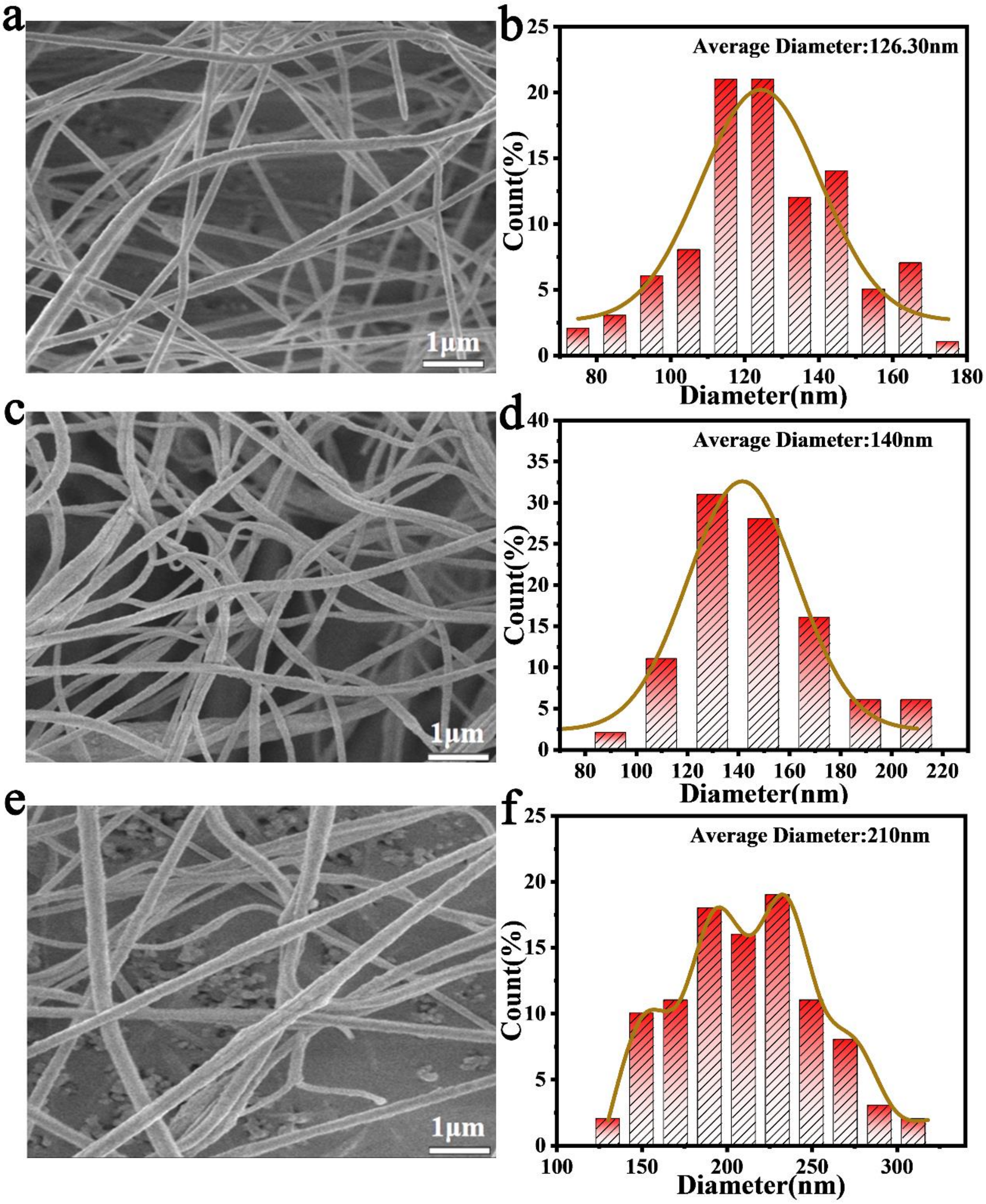

Figure S3. SEM images and diameter distribution of (a-b) S-1, (c-d) S-2, (e-f) S-5.

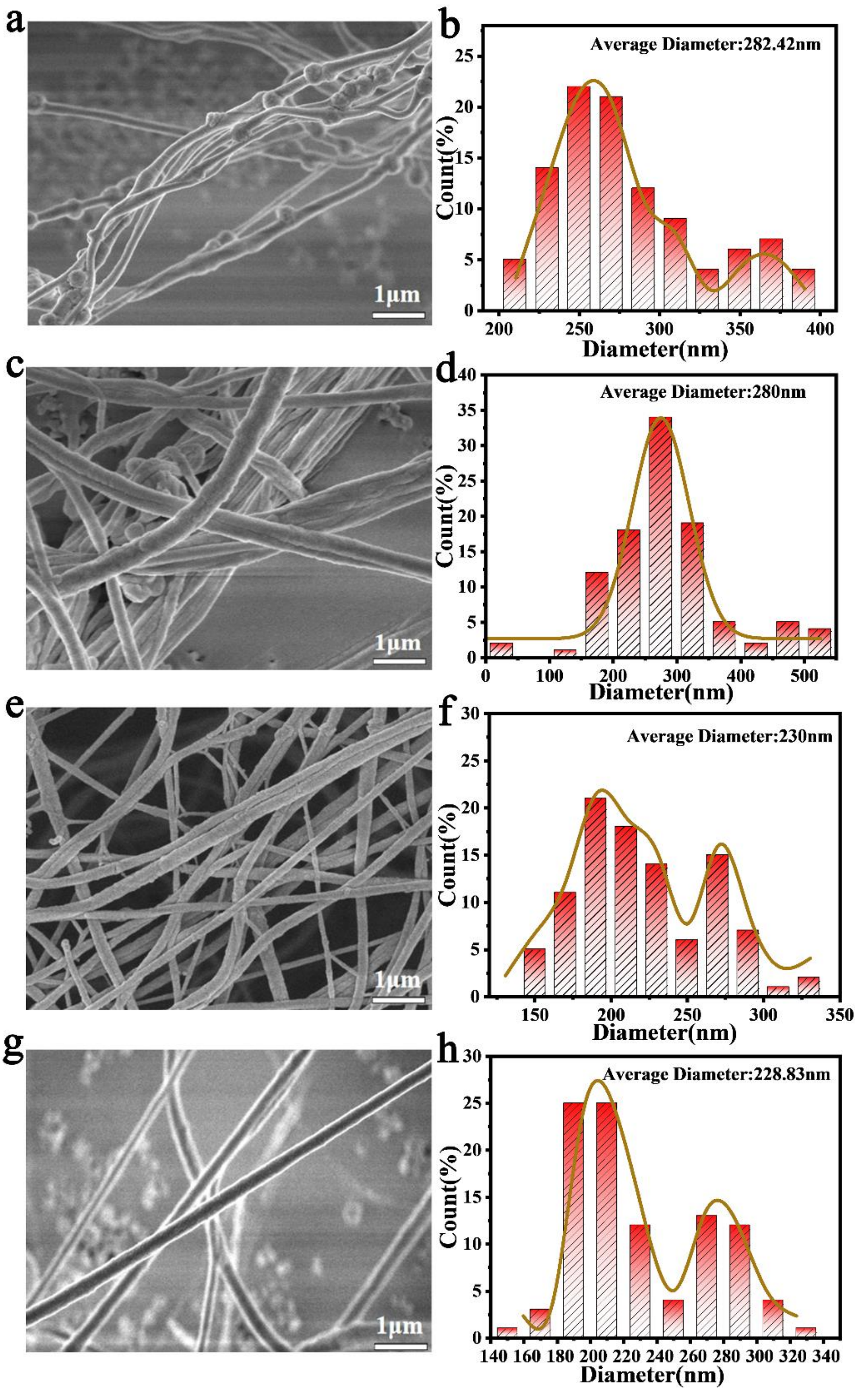

Figure S4. SEM images and diameter distribution of (a-b) PVDF/PDA-1, (c-d) PVDF/PDA-2, (e-f) PVDF/PDA-3, (g-h) PVDF/PDA-5.

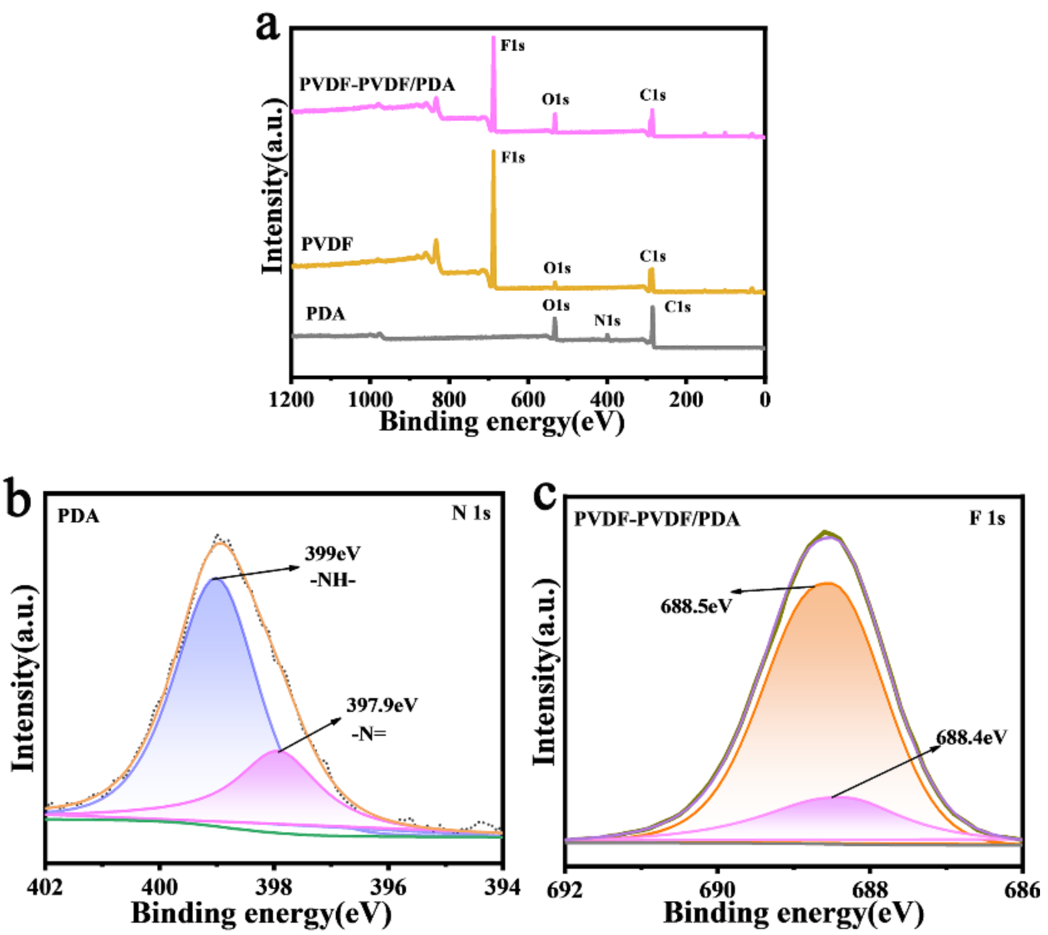

Figure S5. (a) XPS full spectrum, and XPS spectra of (b) N1s for PDA and (c) F1s for PVDF-PVDF/PDA NFMs.

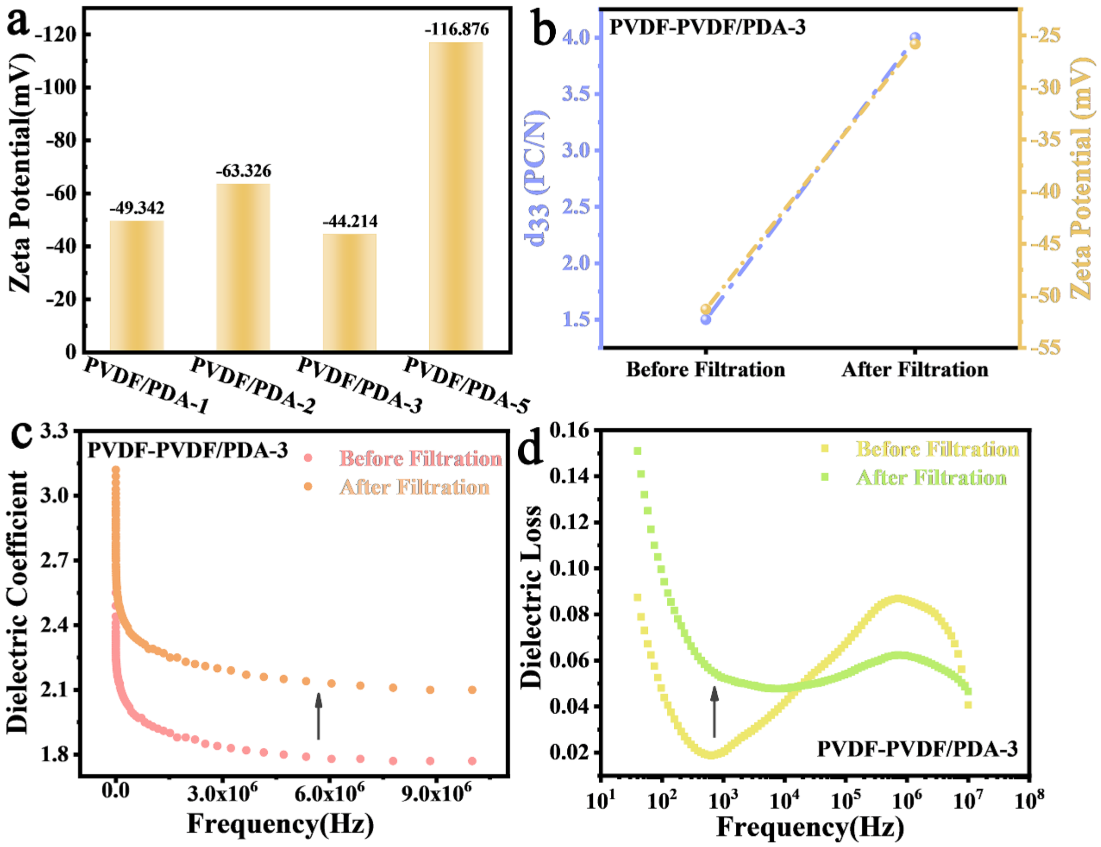

Figure S6. (a) Zeta potential on the solid surface of PVDF/PDA NFMs. (b) Changes in d_{33} and zeta potential on the solid surface of PVDF-PVDF/PDA-3 NFMs after filtration. (c-d) Changes in dielectric coefficient and dielectric loss of PVDF-PVDF/PDA NFMs after filtration.

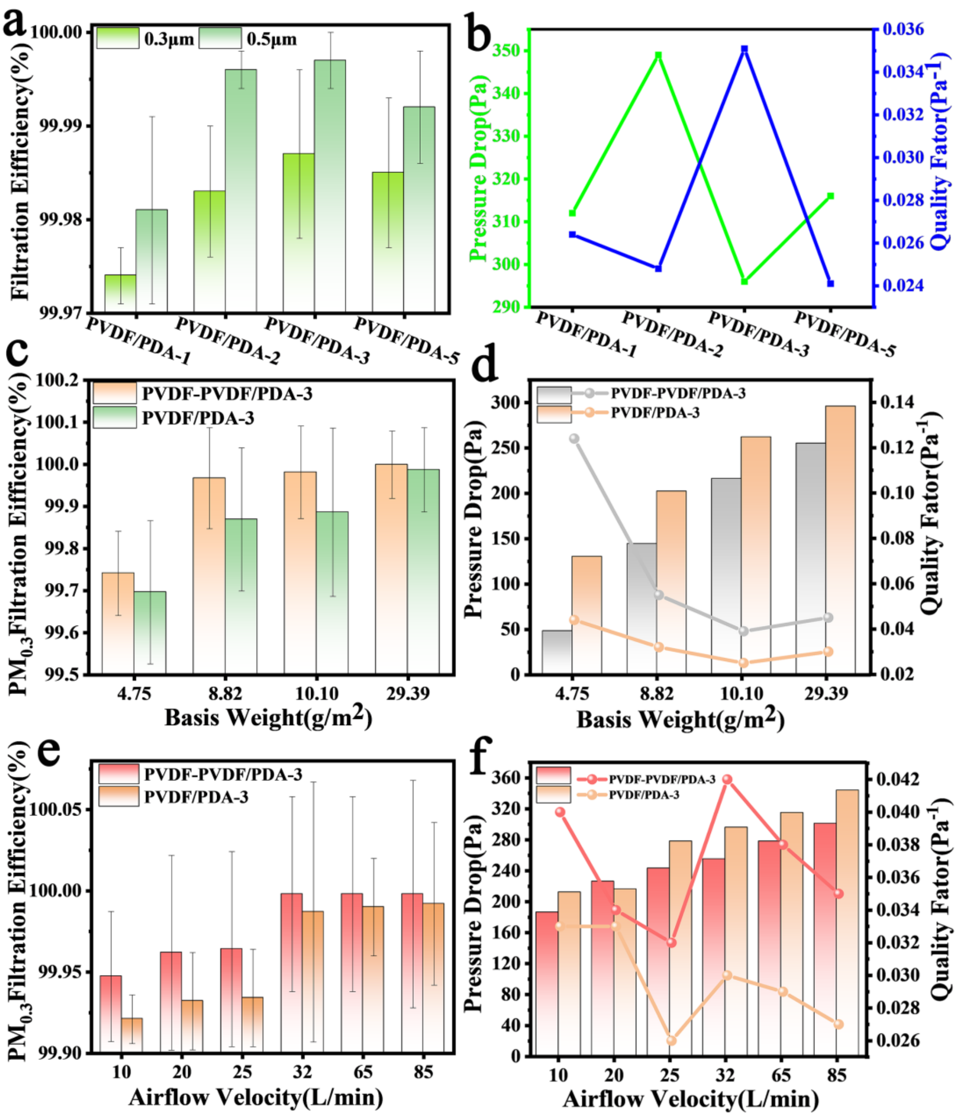

Figure S7. (a-b) PMs filtration efficiency, pressure drop and quality factors of PVDF/PDA NFMs. $PM_{0.3}$ filterability of PVDF-PVDF/PDA-3 and PVDF/PDA-3 NFMs with (c-d) different base weights and (e-f) different airflow velocities.

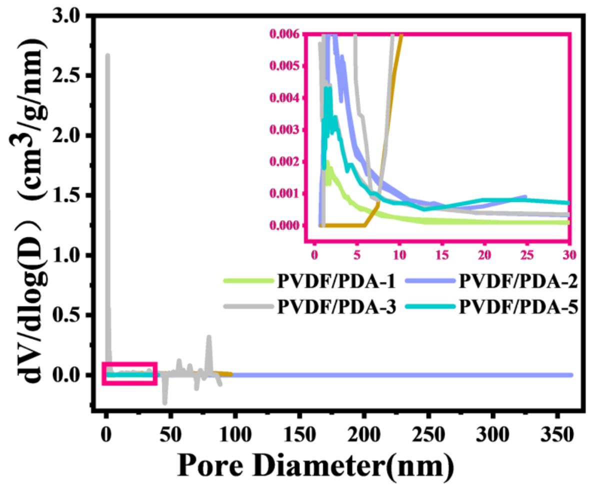

Figure S8. Pore diameter distributions of PVDF/PDA NFMs.

Table S1. Specific surface area, total pore volume and microspore volume of PVDF/PDA NFMs with different PDA doping amounts

Samples	Specific Area (m^2/g)	Total pore volume (cm^3/g)	Microspore volume (cm^3/g)
PVDF/PDA-1	12.218	0.06491	0.00344
PVDF/PDA-2	16.715	0.08802	0.00401
PVDF/PDA-3	16.928	0.09843	0.00483
PVDF/PDA-5	8.602	0.03713	0.00271

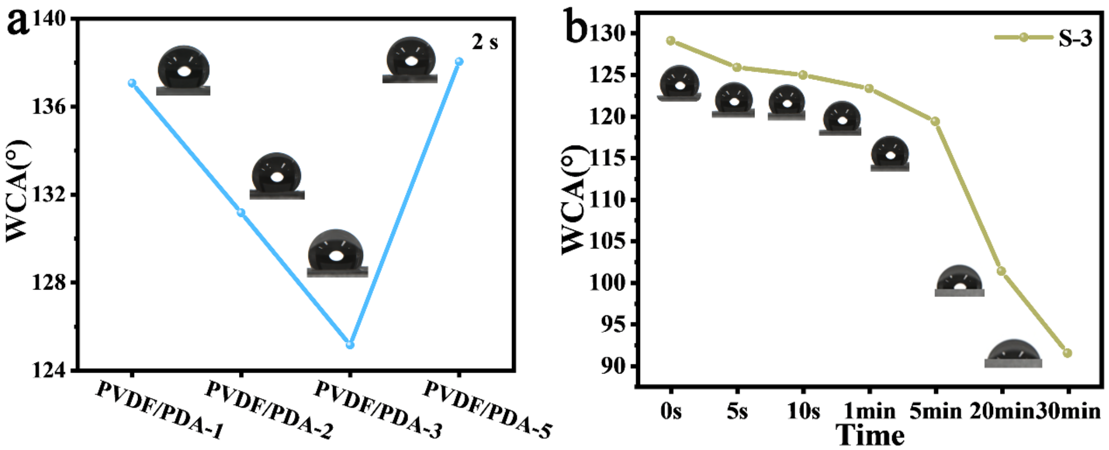

Figure S9. (a) Water contact angle of PVDF/PDA-x NFMs. (b) Water contact angle changes with time of S-3.

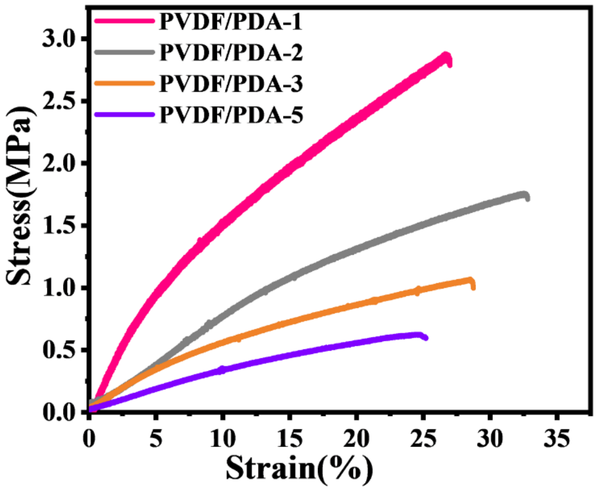

Figure S10. Stress-strain curves of PVDF/PDA-x NFMs.

Table S2. Nanofiber membrane various performance comparison from the reported literature

Material	Diameter (nm)	*H* (%)	*ΔP* (Pa)	*QF* (Pa^{-1})	Electrical performance	Flame resistance	Ref.
F-PVDF-HFP/SiO_2	70	99.59	131.722	0.0417	×	×	[1]
VDF/Nylon	694	92.7	86	0305	√	×	[2]
P(VDF-TrFE)/$BaTiO_3$	200	96	182	0.0250	√	×	[3]
GO/PVDF	551	99.31	35	0.142	√	×	[4]
PVDF/DMAc/ACE	631.7	99.5	27.4	0.153	×	×	[5]
PUL/PVDF	180	98.52	39	0.1080	×	×	[6]
PVDF/DMAc/MEK	70	97.40	51	0.0700	√	×	[7]
PVDF/PMMA/SiO_2	195.16	96.39	44.88	0.0740	×	×	[8]
PVDF-*g*-POEM	77.53	93.56	49	0.0559	×	×	[9]
PVDF-HFP	200-800	98	64	0.0611	√	×	[10]
PVDF/ SiO_2/Ag	270.72	99.94	112.3	0.066	×	×	[11]
PVDF/ZIF-8	140-523	99.90	71	0.170	×	×	[12]
PVDF/PEG	50-170	99.57	158	0.0345	×	×	[13]
PVDF/Fe_3O_4	260	99.95	58.5	0.130	√	×	[14]
PVDF-PVDF/PDA	146.42	99.967	144	0.0556	√	√	★

References

1. Sanyal, A.; Sinha-Ray, S., Ultrafine PVDF nanofibers for filtration of air-borne particulate matters: a comprehensive review. *Polymers* **2021,** *13* (11), 1864.
2. Peng, Z.; Shi, J.; Xiao, X.; Hong, Y.; Li, X.; Zhang, W.; Cheng, Y.; Wang, Z.; Li, W. J.; Chen,

J.; Leung, M. K. H.; Yang, Z., Self-charging electrostatic face masks leveraging triboelectrification for prolonged air filtration. *Nat. Commun.* **2022,** *13* (1), 7835.

3. Su, C.; Zhang, L.; Zhang, Y.; Huang, X.; Ye, Y.; Xia, Y.; Gong, Z.; Qin, X.; Liu, Y.; Guo, S., P(VDF-TrFE)/$BaTiO_3$ nanofibrous membrane with enhanced piezoelectricity for high $PM_{0.3}$ filtration and reusable face masks. *ACS Appl. Mater. Interfaces* **2023,** *15* (4), 5845-5855.
4. Chen, M.; Jiang, J.; Feng, S.; Low, Z.-X.; Zhong, Z.; Xing, W., Graphene oxide functionalized polyvinylidene fluoride nanofibrous membranes for efficient particulate matter removal. *J. Membr. Sci.* **2021,** *635*, 119463.
5. Al-Attabi, R.; She, F.; Zhao, S.; Dumée, L. F.; Schütz, J. A.; Xing, W.; Zhong, Z.; Kong, L., Durable and comfortable electrospun nanofiber membranes for face mask applications. *Sep. Purif. Technol.* **2023,** *322*, 124370.
6. Wang, H.; Bao, Y.; Yang, X.; Lan, X.; Guo, J.; Pan, Y.; Huang, W.; Tang, L.; Luo, Z.; Zhou, B.; Yao, J.; Chen, X., Study on filtration performance of PVDF/PUL composite air filtration membrane based on far-field electrospinning. *Polymers* **2022,** *14* (16), 3294.
7. Bui, T. T.; Shin, M. K.; Jee, S. Y.; Long, D. X.; Hong, J.; Kim, M.-G., Ferroelectric PVDF nanofiber membrane for high-efficiency $PM_{0.3}$ air filtration with low air flow resistance. *Colloids Surf., A* **2022,** *640*, 128418.
8. Zheng, J.; Zhou, X.; Wang, B.; Dai, F.; Liu, J., Modified PVDF/PMMA/SiO_2 composite nanofibrous membrane in airborne filtration: transparency, mechanical properties and filtration performance. *J. Environ. Chem. Eng.* **2024,** *12* (6), 114109.
9. Moon, J.; Bui, T. T.; Jang, S.; Ji, S.; Park, J. T.; Kim, M.-G., A highly efficient nanofibrous air filter membrane fabricated using electrospun amphiphilic PVDF-g-POEM double comb copolymer. *Sep. Purif. Technol.* **2021,** *279*, 119625.
10. Gao, H.; Li, Z.-J.; Xu, X.-F.; Wang, N.; Yang, M.-Y.; Long, Y.-Z.; Zhang, H.-D., Electrospinning dual energy-saving design of PVDF-HFP nanofiber films for passive radiant cooling and air filtration. *AIP Adv.* **2024,** *14* (1), 015349
11. Wu, Y.; Li, X.; Zhong, Q.; Wang, F.; Yang, B., Preparation and filtration performance of antibacterial PVDF/SiO_2/Ag composite nanofiber membrane. *Journal of Building Engineering* **2023,** *74*, 106864.
12. Geng, Q.; Dong, S.; Li, Y.; Wu, H.; Yang, X.; Ning, X.; Yuan, D., High-performance photoinduced antimicrobial membrane toward efficient $PM_{2.5-0.3}$ capture and oil-water separation. *Sep. Purif. Technol.* **2022,** *284*.
13. Toptaş, A.; Çalışır, M. D.; Kılıç, A., Production of ultrafine PVDF nanofiber/nanonet-based air filters via the electroblowing technique by employing PEG as a pore-forming agent. *ACS Omega* **2023,** *8* (41), 38557-38565.
14. Liu, F.; Li, M.; Li, F.; Weng, K.; Qi, K.; Liu, C.; Ni, Q.; Tao, X.; Zhang, J.; Shao, W.; He, J., Preparation and properties of PVDF/Fe_3O_4 nanofibers with magnetic and electret effects and their application in air filtration. *Macromol. Mater. Eng.* **2020,** *305* (4), 1900856.
